# Electrolyte prognosis scoring system can predict overall survival in patients with osteosarcoma

**DOI:** 10.3389/fonc.2024.1466912

**Published:** 2024-10-09

**Authors:** Han Liu, Hui Kang, Longqing Li, Zhuangzhuang Li, Xuanhong He, Yuqi Zhang, Minxun Lu, Li Min, Chongqi Tu

**Affiliations:** ^1^ Department of Orthopedics, Orthopaedic Research Institute, West China Hospital, Sichuan University, Chengdu, China; ^2^ Department of Operating Room, West China Hospital, Sichuan University/Nursing Key Laboratory of Sichuan Province, Sichuan University, Chengdu, China

**Keywords:** osteosarcoma, hematological markers, prognostic nomograms, electrolyte, OS

## Abstract

Osteosarcoma stands as the most prevalent bone tumor, characterized by a heightened tendency for local recurrence and distant metastasis, resulting in a bleak prognosis. Presently, there exists a shortage of novel markers to effectively determine the prognosis of osteosarcoma patients. Recent research indicates that hematological markers partially mirror an individual’s microenvironment, offering potential insights into predicting patient prognosis. However, prior studies predominantly focused on the prognostic significance of singular hematological indices, failing to comprehensively represent the tumor microenvironment of patients. In our investigation, we meticulously gathered data on 22 hematological and electrolyte markers, utilizing LASSO Cox regression analysis to devise an Electrolyte Prognostic Scoring System (EPSS). The EPSS encompasses various indicators, including immunity, inflammation, coagulation, and electrolyte levels. Our findings indicate that the EPSS stands as an independent prognostic factor for overall survival among osteosarcoma patients. It serves as a valuable addition to clinical characteristics, adept at discerning high-risk patients from those deemed clinically low-risk. Furthermore, EPSS-based nomograms demonstrate commendable predictive capabilities.

## 1 Introduction

Osteosarcoma, comprising 20% to 40% of all bone tumors (1–3), stands as the most prevalent bone tumor with a pronounced propensity for local recurrence and distant metastasis, resulting in a dismal prognosis. The introduction of chemotherapy treatment in the 1970s notably augmented the five-year survival rate for patients with nonmetastatic osteosarcoma (4). As comprehensive treatments advance, the 5-year overall survival (OS) rate has improved to 60%–70%. However, this rate decreases significantly to 20%–30% when metastasis occurs. At the time of presentation, around 15%–20% of affected patients already exhibit metastases, and those with metastatic disease face notably low short- and long-term survival rates (5–8). Furthermore, tumor recurrence and the development of chemoresistance are recognized as crucial prognostic factors (9, 10). These clinical characteristics play a pivotal role in identifying high-risk patients and guiding treatment decisions (11). Nonetheless, disease progression might vary among patients sharing similar clinical traits. Hence, it becomes imperative to consider additional factors to enable precise and tailored treatment approaches.

Recent studies have revealed that certain preoperative hematological markers, such as the neutrophil-to-lymphocyte ratio (NLR), platelet-to-lymphocyte ratio (PLR), LDH, HBDH, and lymphocyte-to-macrophage ratio (LMR), offer insights into an individual’s tumor microenvironment, and these markers have shown promise in predicting the prognosis of cancer patients (12–16). They are easily accessible, cost-effective, and serve as valuable prognostic indicators. Numerous recent studies have underscored their significance in predicting survival and treatment response in cancer, including osteosarcoma (17, 18). Moreover, recent literature suggests that the presence of hyponatremia, hypochloremia, hypocalcemia, and hyperuricemia correlates with poorer survival rates. Surprisingly, there remains a dearth of studies investigating the relationship between electrolyte levels and the prognosis of osteosarcoma (19).

The LASSO model is an estimation method that enables the reduction of the set of indicators. LASSO regression has the advantages of ridge regression and subset selection at the same time, which makes it superior to other methods in terms of prediction accuracy and model interpretability for high dimensional multicollinearity problems. These advantages make LASSO regression an important tool in cancer biomarker research and prognostic model construction. In our study, we gathered established prognostic hematologic and electrolyte markers, utilizing iterative least absolute contraction and selection operator (LASSO) COX proportional hazards regression analysis to devise the Electrolyte Prognostic Scoring System (EPSS). Our findings indicate that EPSS addresses drawbacks inherent in single hematological markers, such as inadequate predictive power and instability, making it a valuable complement to clinical features.

## 2 Methods

### 2.1 Patients

**Figure 1:**
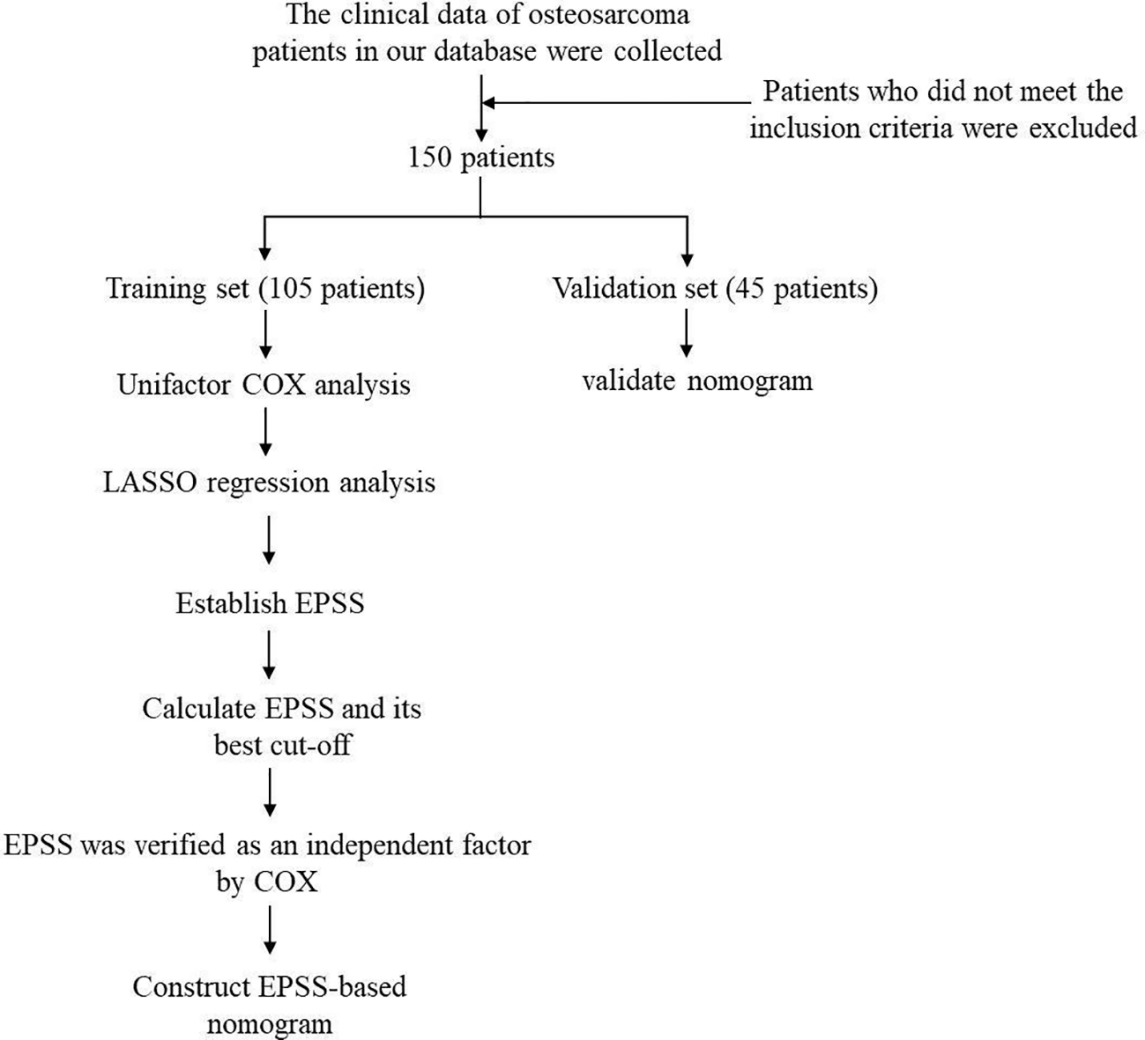
Work flow chart of this study.

The flow chart through this study is presented in **Figure 1**. With the approval of the Medical Ethics Committee, we reviewed the clinical data of osteosarcoma patients from January 2012 to January 2022 in the database of the Musculoskeletal Tumor Center of West China Hospital. During the review process, we included and excluded patients according to the following criteria: 1) patients with high grade osteosarcoma confirmed by histopathology; 2) patients have complete hematological test results before neoadjuvant chemotherapy; 3) patient received standard treatment at West China Hospital. The exclusion criteria:1) Patients with histopathologically confirmed low-grade osteosarcoma (intramedullary and bone surface) and periosteal osteosarcoma; 2) Patients who had received neoadjuvant chemotherapy before their first-time consultancy in our hospital; 3) patients with hematological diseases; 4) patients with other malignancies; 5) patients not received standard treatment (patients who are misdiagnosed and mistreated or fail to complete postoperative chemotherapy). Finally, 150 patients were included in our study after passing the inclusion and exclusion criteria. Each patient was followed up regularly until death or January 2022. The following follow-up principles were followed: reexamination every 3 months within 1 year after surgery; reexamination every 4 months 1-2 years after surgery; reexamination every 5 months 2-3 years after surgery; reexamination every 6 months 3-5 years after surgery; reexamination every year more than 5 years after surgery. All patients were randomly divided into a training set (n=105, 70%) and external validation set (n=45, 30%) using a random seed set in 2022.

### 2.2 Date collection and processing

We gathered various hematological and biochemical parameters from the initial blood routine, coagulation function tests, and liver and kidney function assessments of 150 patients before neoadjuvant chemotherapy. These included Hemoglobin (HB), Platelet count (Plt), Leukocyte count (Leut), Neutrophilic granulocyte percentage (Neup), Lymphocyte percentage (LYMp), Neutrophil count (Neut), Lymphocyte count (LYMPH), Monocyte count (MONO), Alanine aminotransferase (ALT), Aspartate aminotransferase (AST), Albumin (Protein), Alkaline phosphatase (ALP), Glutamyl transpeptidase (Glu), Creatine kinase (CK), Lactate dehydrogenase (LDH), Hydroxybutyrate dehydrogenase (HBDH), Natrium ion (Na), Anion gap (AG), Serum beta-hydroxybutyric acid (serum β), and Phosphorus (P).

Formulas were employed to compute several ratios including Neutrophil-to-Lymphocyte Ratio (NLR), Platelet-to-Lymphocyte Ratio (PLR), Lymphocyte-to-Monocyte Ratio (LMR), and derived Neutrophil-to-Leukocyte Ratio (dNLR), determined as follows: NLR = Neut/LYMPH, PLR = PLT/LYMPH, LMR = LYMPH/MONO, and dNLR = Neut/(Leut-Neut). Additionally, patient demographics such as age, gender, tumor location, and pathological fracture status were abstracted from medical records.

Overall survival (OS) was calculated from the date of tumor resection until the last follow-up or date of death. Optimal cutoff values for each hematological marker were determined using time-dependent receiver operating curve (tdROC) analysis, which were then converted into binary variables based on these cutoff values in the overall patient cohort.

### 2.3 Establishment and validation of EPSS (electrolyte prognostic scoring system)

We initiated our analysis by employing univariate Cox regression analysis to identify potential prognostic indicators within the overall patient cohort. Subsequently, leveraging the identified prognostic hematological markers, we conducted LASSO Cox regression analysis on the training set to ascertain the optimal Hematological Prognostic Scoring System (EPSS). EPSS scores were computed for each patient in both the training and validation sets based on coefficients derived from the LASSO Cox regression analysis.

To assess the predictive performance of EPSS compared to individual hematological markers, receiver operating curves (ROCs) were utilized in both the training and validation sets. Within the training set, the optimal cutoff value for EPSS was determined using the survival-ROC package, subsequently stratifying patients into high-risk and low-risk groups based on this cutoff value, which was then applied to the validation set. Kaplan-Meier survival curves were generated to visualize the disparity in Overall Survival (OS) between the two groups of patients.

The independent prognostic capacity of EPSS in predicting OS among osteosarcoma patients was evaluated using multivariate Cox regression analysis. Additionally, ROC curves were plotted for EPSS and clinical variables, spanning from 1 to 5 years, in both the training and validation sets using the time-ROC package, allowing for comparison.

### 2.4 Construction and evaluation of the nomogram

A nomogram was developed by combining EPSS with clinical features within the training set. Harrell’s Concordance Index was utilized to evaluate the nomograms’ discrimination ability, while calibration curves were employed to assess accuracy. Decision curve analysis (DCA) was conducted to appraise the clinical usefulness of the nomogram. Additionally, the established nomogram was applied to forecast overall survival in the validation cohort, affirming the stability and predictive capacity of the model.

### 2.5 Statistical analysis

The Kolmogorov–Smirnov test was used to assess whether continuous variables were normally distributed, and the Mann-Whitney U test or Spearman correlation analysis was used to assess differences between continuous variables according to the results. Categorical variables were evaluated using the chi-square test and Fisher’s exact test based on the number of individuals in each group. All statistical analyses were conducted using R software, version 4.3.1 (Institute for Statistics and Mathematics, Vienna, Austria). P-values < 0.05 were considered to indicate statistical significance.

## 3 Results

### 3.1 Patient characteristics

The study encompassed a cohort of 150 osteosarcoma patients, comprising 95 males and 55 females. Patients’ ages ranged from 8 to 72 years, with a mean age of 24.9 years. The majority of patients had tumors located in the extremities, while only 12 patients presented with tumors in non-extremity sites. Six patients had pathological fractures upon presentation. The 150 patients were randomly divided into a training cohort and a validation cohort. **Table 1** displays the demographic and clinical characteristics of both the training and validation cohorts. No significant differences were observed between the two groups in terms of these characteristics. **Supplementary Table 1** includes the optimal cutoff values for 22 hematological and electrolyte markers (HB, Plt, Leut, Neup, LYMp, Neut, LYMPH, MONO, NLR, PLR, LMR, dNLR, Protein, ALP, Glu, CK, LDH, HBDH, Na, AG, serumβ, P). Notably, the distributions of all variables in both the training and validation sets showed no significant differences, as demonstrated in **Table 1**.

**Table 1:** Characteristics of 150 osteosarcoma patients.

Characteristics	Train(n=105)	Test(n=45)	P-value	Coefficient
OS				Not applicable
Alive	75(71.4%)	29(64.4%)	0.4417	
Died	30(28.5%)	16(35.6%)		
Gender				Not applicable
Male	67(63.8%)	28(62.2%)	1	
Female	38(36.2%)	17(37.8%)		
Age(years)				Not applicable
Mean	23.8	27.7	0.3012	
Tumor-site				Not applicable
Extremities	97(92.4%)	41(91.1%)	1	
Non-extremities	8(7.6%)	4(8.9%)		
Pathological-fracture				Not applicable
No	101(96.2%)	43(95.6%)	1	
Yes	4(3.8%)	2(4.4%)		
HB				-0.5452673
Low	42(40%)	17(37.8%)	0.8565	
High	63(60%)	28(62.2%)		
Plt				0.5519471
Low	31(29.5%)	14(31.1%)	1	
High	74(70.5%)	31(68.9%)		
Leut				Not applicable
Low	42(40%)	17(37.8%)	0.8565	
High	63(60%)	28(62.2%)		
Neup				0.2702047
Low	41(39%)	14(31.1%)	0.4218	
High	64(61%)	31(68.9%)		
LYMp				Not applicable
Low	67(63.8%)	30(66.7%)	1	
High	38(36.8%)	15(33.3%)		
Neut				Not applicable
Low	62(59%)	27(60%)	1	
High	43(41%)	18(40%)		
LYMPH				Not applicable
Low	43(41%)	15(33.3%)	0.4651	
High	62(59%)	30(66.7%)		
MONO				Not applicable
Low	73(69.5%)	29(64.4%)	0.5701	
High	32(30.5%)	16(35.6%)		
NLR				Not applicable
Low	68(64.8%)	30(66.7%)	0.8538	
High	37(35.2%)	15(33.3%)		
PLR				Excluded
Low	68(64.8%)	27(60%)	0.7119	
High	37(35.2%)	18(40%)		
LMR				Not applicable
Low	52(49.5%)	30(66.7%)	0.5968	
High	53(50.5%)	25(55.6%)		
dNLR				0.3582851
Low	41(39%)	14(31.1%)	0.4599	
High	64(61%)	31(68.9%)		
ALT				Not applicable
Low	17(16.1%)	6(13.3%)	0.8063	
High	88(83.9%)	39(86.7%)		
AST				Not applicable
Low	9(8.6%)	4(8.9%)	1	
High	96(91.4%)	41(91.1%)		
Protein				Not applicable
Low	39(37.1%)	15(33.3%)	0.7131	
High	66(62.9%)	30(66.7%)		
ALP				0.1939839
Low	30(28.6%)	19(42.2%)	0.1288	
High	75(71.4%)	26(57.8%)		
Glu				Not applicable
Low	93(88.6%)	35(77.8%)	0.1285	
High	12(11.4%)	10(22.2%)		
CK				Not applicable
Low	17(16.2%)	6(13.3%)	0.8063	
High	88(83.8%)	39(86.7%)		
LDH				Not applicable
Low	54(51.4%)	25(55.6%)	0.7222	
High	51(48.6%)	20(44.4%)		
HBDH				Not applicable
Low	84(80%)	36(80%)	1	
High	21(20%)	9(20%)		
Na				-0.7019458
Low	88(83.8%)	36(80%)	0.6393	
High	17(16.2%)	9(20%)		
AG				0.6730434
Low	65(61.9%)	27(60%)	0.8562	
High	40(38.1%)	18(40%)		
serumβ				Not applicable
Low	74(70.5%)	30(66.7%)	0.7005	
High	31(29.5%)	15(33.3%)		
P				0.5723482
Low	24(22.9%)	15(33.3%)	0.2232	
High	81(77.1%)	30(66.7%)		

### 3.2 Establishment and validation of electrolyte risk model for osteosarcoma

**Figure 2:**
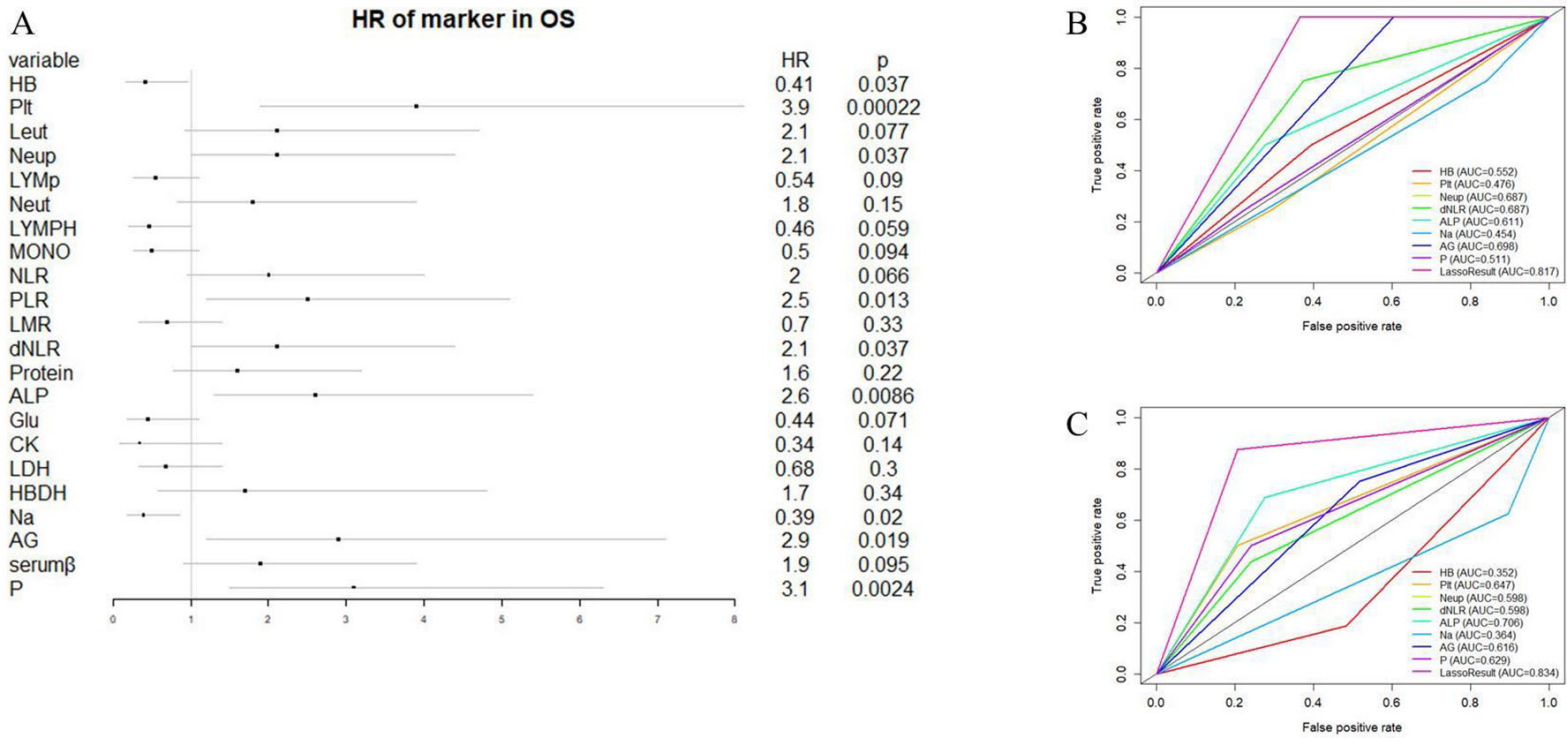
Construction of EPSS and its comparison with individual hematological and electrolyte parameters. **(A)** Forest plot showing the results of univariate cox regression analysis of 22 hematological and electrolyte markers; **(B)** ROC curves showing the predictive power of EPSS in the training set versus a single hematology or electrolyte indicator; **(C)** ROC curves showing the predictive power of EPSS in the validation set versus a single hematology or electrolyte indicator.

We initiated our study with univariate Cox regression analysis, evaluating the association between hematologic and electrolyte markers with OS in osteosarcoma patients within the overall cohort. **Figure 2A** illustrates that nine hematological markers demonstrated statistical significance in the univariate Cox regression analysis. Subsequently, employing LASSO Cox regression analysis in the training set using these nine hematological indicators, we identified an EPSS composed of eight significant hematological indicators (**Table 1**). The risk formula can be expressed as: Riskscore=HB*(-0.56) +Plt*0.55+Neup*0.27+dNLR*0.36+ALP*0.19+Na*(-0.70) +AG*0.67+P*0.57.

The coefficients assigned to each indicator in the EPSS are detailed in **Table 1**, facilitating the calculation of EPSS for each patient based on these coefficients. Our ROC curve analysis demonstrated a notably enhanced predictive ability of EPSS compared to individual hematological markers in both the training and validation cohorts (0.817 vs 0.454-0.698; 0.834 vs 0.352-0.706, **Figures 2B, C**).

**Figure 3:**
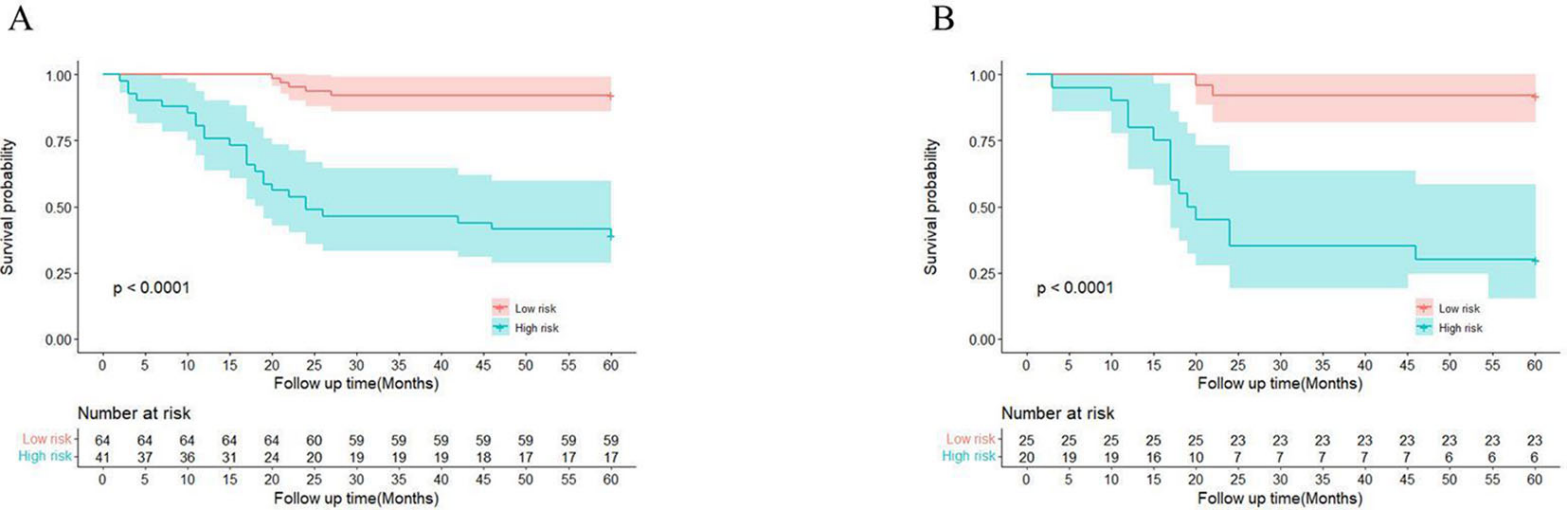
There are significant differences between patients in EPSS risk groups. **(A)** High-risk patients in the training set had significantly lower overall survival than low-risk patients; **(B)** High-risk patients in the validation set had significantly lower overall survival than low-risk patients.

We also determined optimal cutoff values for EPSS, stratifying the training cohort and validating it in the validation cohort. As depicted in **Figures 3A, B**, patients in the high EPSS risk group exhibited significantly lower overall survival rates in both cohorts (P < 0.001).

**Figure 4:**
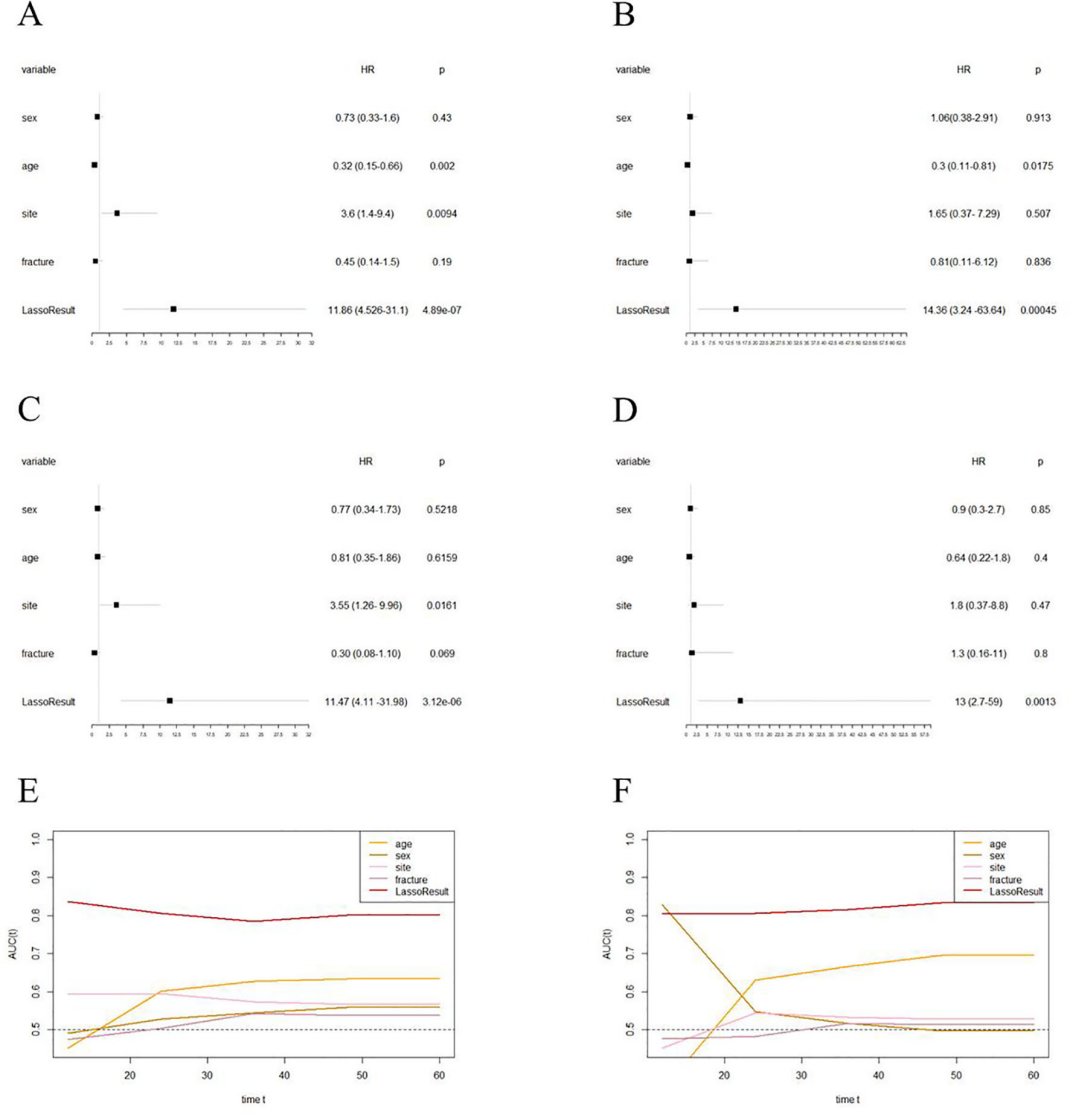
EPSS is an independent prognostic factor for overall survival in patients with osteosarcoma and has certain advantages compared with clinical characteristics. **(A)** Forest plot showing the results of univariate COX regression analysis of EPSS and clinical characteristics in the training set; **(B)** Forest plot showing the results of univariate COX regression analysis of EPSS and clinical characteristics in the validation set; **(C)** Forest plot showing the results of multivariate COX regression analysis of EPSS and clinical characteristics in the training set; **(D)** Forest plot showing the results of multivariate COX regression analysis of EPSS and clinical characteristics in the validation set; **(F)** Time-dependent ROC curves showing the predictive power of EPSS and clinical features in the training set; **(F)** Time-dependent ROC curves showing the predictive power of EPSS and clinical features in the training set; It can be seen that the predictive power of each variable varies over time.

Further analysis through multivariate Cox regression confirmed EPSS as an independent prognostic factor for overall survival in osteosarcoma patients within both the training and validation cohorts (training cohort: HR: 11.47(4.11-31.98); validation cohort: HR: 13(2.7-59), **Figure 4A–D**).

Lastly, we plotted time-dependent ROC curves to compare the predictive ability of EPSS with clinical features such as tumor site, metastatic status, and pathological fractures. **Figures 4E, F** illustrate similar predictive abilities of EPSS over time in both the training and validation cohorts, indicating a gradual improvement in its predictive capacity.

### 3.3 Construction and validation of EPSS-based nomograms

**Figure 5:**
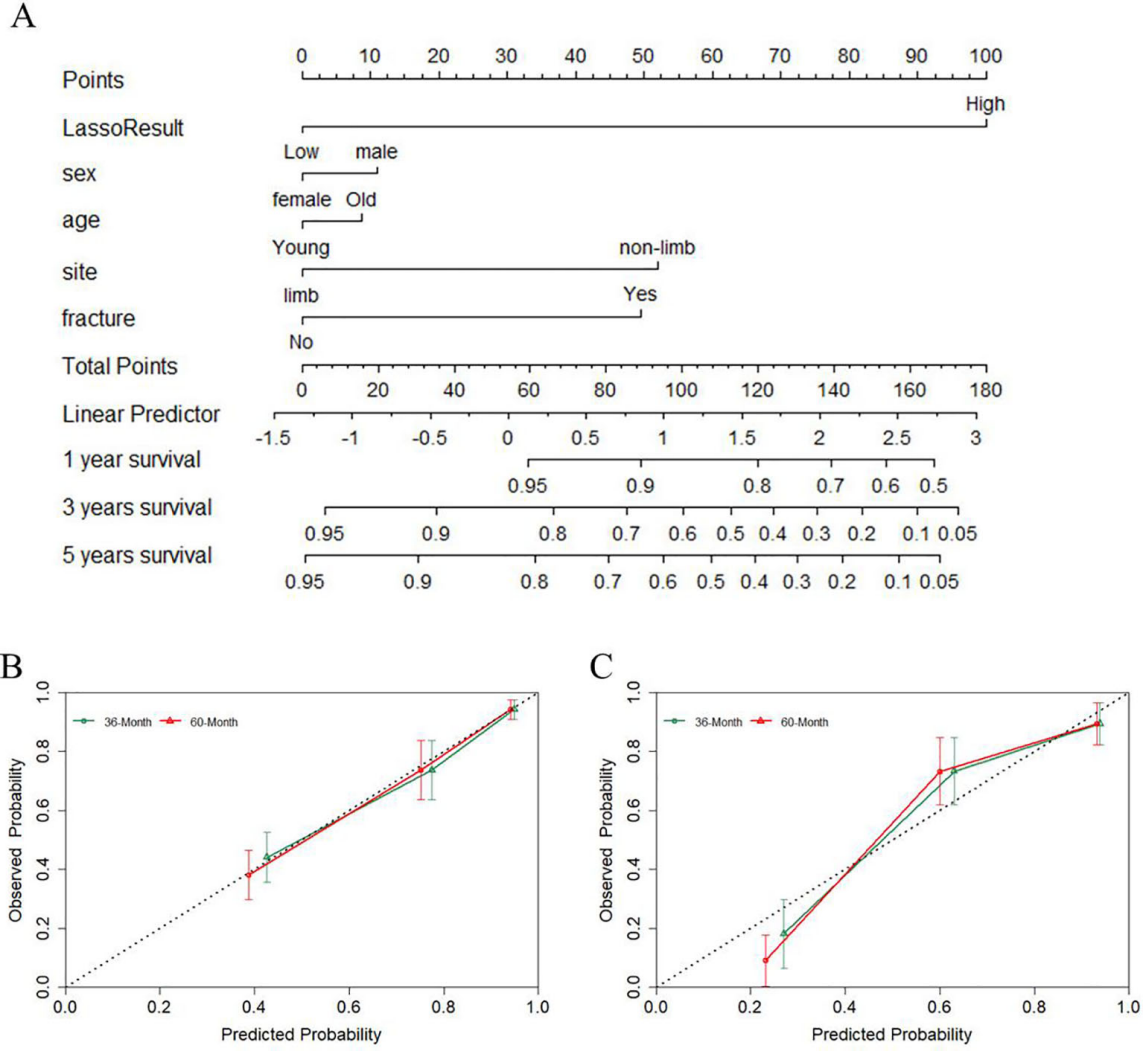
A nomogram was constructed combining EPSS with clinical features and the predictive power of the nomogram was assessed. **(A)** The nomogram of the overall survival of patients with osteosarcoma shows that EPSS score and site are the two most important variables; **(B)** Calibration curves for nomogram predicting 36-Month and 60-Month survival of patients in the training set; **(C)** Calibration curves for nomogram predicting 36-Month and 60-Month survival of patients in the validation set.

To facilitate the clinical applicability of EPSS, we developed a nomogram by integrating EPSS with clinical characteristics based on the training cohort. Cox proportional hazards regression assigned a score corresponding to the hazard ratio for each covariate in the nomogram. The cumulative scores of these covariates constituted the nomogram’s total score. With a C-index of 0.78, the constructed nomogram exhibited good predictive accuracy for 36-month and 60-month overall survival in the training cohort, as depicted in **Figures 5A** and **5B** through calibration curves.

To validate the robustness of the nomogram, we assessed its performance in the validation cohort. With a C-index of 0.79, the nomogram displayed commendable predictive ability in the validation set, as indicated by the calibration curve in **Figure 5C**.

### 3.4 Association between EPSS and clinical features

**Figure 6:**
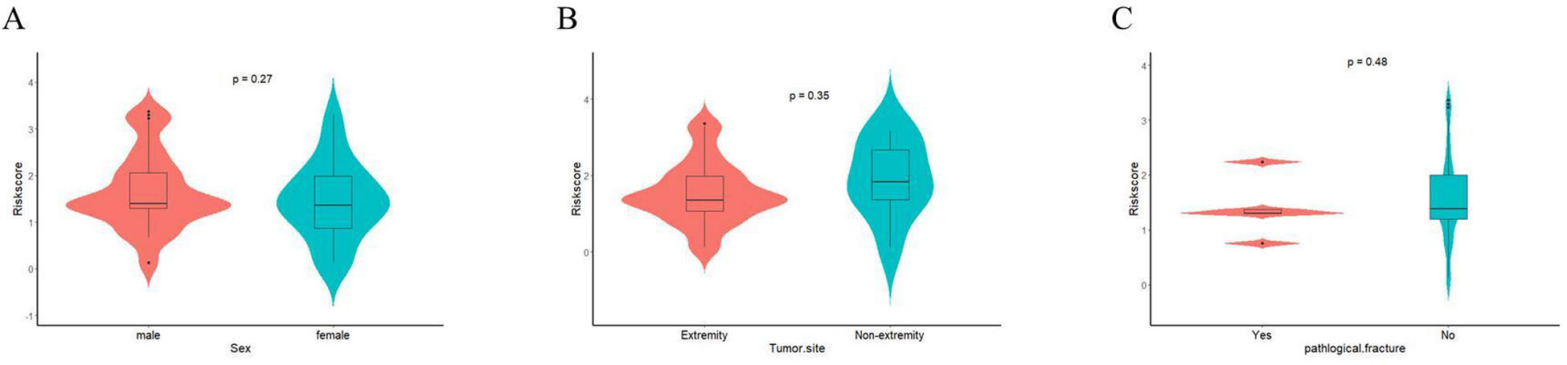
The relationship between EPSS and clinical characteristics were assessed. **(A)** The relationship between EPSS and gender; **(B)** The relationship between EPSS and Tumor-site; **(C)** The relationship between EPSS and pathological fracture.

Finally, we further assessed the relationship between EPSS and clinical characteristics. The results of the violin plot indicated that there was no significant difference in EPSS among patients with different gender, tumor site and pathological fracture (**Figures 6A–C**).

## 4 Discussion

As surgical techniques advance and novel treatments emerge, the mortality rates among cancer patients have shown a declining trend. However, despite these advancements, the overall survival rates for osteosarcoma patients have plateaued since the 1970s (1, 11, 20). The evolution of precision medicine emphasizes the imperative need for tailored treatment strategies, offering the potential to enhance patient prognosis through individualized management (21, 22). Growing evidence underscores the pivotal role of genetic changes and epigenetic modifications in tumor onset and progression. Genetic testing, particularly assessing response to drug therapy, has gained traction in clinical applications. However, many of these tests rely on patient tissue and are cost-intensive. Encouragingly, recent research has unveiled that several preoperative hematological markers hold promise in predicting the prognosis of cancer patients (23–28). Contrary to genetic testing, hematological markers offer cost-effective and easily accessible prognostic insights. Remarkably, these markers originate from routine tests conducted upon patient admission. Prior studies often highlighted the prognostic value of singular hematological markers in cancer patients (29–31). However, due to the intricate nature of the tumor microenvironment, a single hematological marker struggles to comprehensively reflect tumor characteristics and accurately predict tumor progression. While previous studies indicated the prognostic value of Lymphocyte-to-Monocyte Ratio (LMR) in osteosarcoma patients, our cohort found LMR to exhibit limited predictive power (16, 32–34). Recent literature highlights associations between electrolyte imbalances (hyponatremia, hypochloremia, hypocalcemia, hyperuricemia) and poorer survival among tumor patients. Surprisingly, there’s a lack of exploration into the relationship between electrolytes and osteosarcoma prognosis.

Our study extensively gathered hematological markers with established prognostic value in osteosarcoma and uniquely combined electrolytes with these markers to construct the EPSS. Compared to singular hematological parameters, EPSS demonstrates superior predictive ability, potentially overcoming the instability of single hematological markers. EPSS demonstrated better predictive power (AUC>0.8) compared with previously published hematological prognostic models that did not include electrolytes (AUC<0.8) (28). Moreover, EPSS surpasses clinical characteristics in predicting long-term patient survival. The EPSS-based nomogram exhibits robust predictive capability, serving as a valuable complement to clinical features. When combined with clinical characteristics, EPSS facilitates further differentiation among patients categorized as clinically low risk.

Inflammation associated with tumors stands recognized as a significant hallmark of cancer. Numerous studies have elucidated how inflammatory processes foster cancer growth, activate oncogenic signaling pathways, and potentially contribute to immune resistance in cancer patients (35, 36). Immune cells, through intricate interactions with cancer cells, exert a pivotal role in shaping the tumor microenvironment (37).While evidence presents a paradoxical role for neutrophils in both impeding and advancing tumor progression, in solid tumors, their proliferation within the tumor microenvironment and systemically often correlates with a poorer prognosis (16, 38, 39). Conversely, lymphocytes in the tumor microenvironment are deemed crucial for antitumor immunity, orchestrating cytokine production and inducing tumor cell apoptosis (40). Platelets play a role in altering the tumor microenvironment by secreting vascular growth factors, promoting tumor cell growth, vascular proliferation, shielding tumor cells from immune cell eradication, and facilitating tumor cell metastasis (41, 42). Lactate dehydrogenase, a classical inflammatory marker in cancer, has undergone extensive study for its prognostic value (15, 43). Notably, within the EPSS framework, Platelet count (Plt) emerged as a cornerstone, exhibiting a coefficient of 0.552. This reaffirms the association between higher Plt levels and poorer patient prognosis, corroborating prior findings.

Serum ALP levels are often positively correlated with osteoblast activity, and serum ALP is common in fractures, physiological growth and bone tumors. Studies on the prognostic value of serum ALP in osteosarcoma date back even before the era of chemotherapy (44, 45). Now, it is generally believed that elevated serum ALP is associated with a worse prognosis in osteosarcoma patients (46). As an important part of constituting the EPSS, the coefficient of ALP was 0.194, indicating that elevated ALP is associated with poor patient prognosis. This is consistent with previous findings. We believe that the introduction of serum ALP makes EPSS more suitable for patients with bone tumors and enhances its predictive ability in patients with bone tumors.

EPSS exhibited superior predictive potency compared to individual hematological markers in both our training and validation cohorts. Our review of previous studies highlighted variations in the prognostic value of different hematological parameters across diverse cohorts, posing challenges in their clinical application. This variability likely stems from the incapacity of single hematological markers to comprehensively address the intricate tumor microenvironment. In response, our study extensively collected multiple hematological and electrolyte markers, culminating in the construction of EPSS to enhance predictive ability. Our aim was to mitigate the inherent instability of individual hematological markers. Notably, EPSS showcased higher predictive power than clinical features. Consequently, we assert EPSS’s effectiveness as a valuable complement to clinical features, particularly in distinguishing high-risk patients within the cohort initially categorized as low-risk based on clinical features.

We recommend utilizing hematological parameters obtained before chemotherapy to calculate EPSS for patients diagnosed with osteosarcoma. These pre-chemotherapy hematological parameters offer a more accurate reflection of the patient’s tumor microenvironment, as the results obtained after chemotherapy might be influenced by the treatment and may not faithfully represent the true tumor microenvironment.

We acknowledge several limitations in our study. Firstly, its retrospective nature may introduce selection bias. Secondly, the composition and calculation of EPSS, involving five hematological and three electrolyte parameters, pose complexity compared to single hematological markers. Notably, the relationship between electrolytes and osteosarcoma prognosis, explored for the first time in this study, lacks previous literature support. Further research is essential to validate our findings. Additionally, exploring whether EPSS can guide osteosarcoma treatment warrants attention. For instance, assessing whether patients with high EPSS, not developing lung metastases, benefit from increased frequency of lung CT follow-ups or if those with high EPSS benefit from increased chemotherapy cycles requires further investigation.

## 5 Conclusions

Our study confirms the prognostic value of the comprehensive hematological score EPSS in patients with osteosarcoma. EPSS is an independent prognostic factor in patients with osteosarcoma. The nomogram constructed based on EPSS has good predictive ability. The EPSS is a valid addition to clinical characteristics and is suitable for further identification of high risk patients from low clinical risk patients.

## Data Availability

The data analyzed in this study is subject to the following licenses/restrictions: nothing. Requests to access these datasets should be directed to xnliuyedao@163,com.
